# Bridging gaps in everyday life – a free-listing approach to explore the variety of activities performed by physiotherapists in specialized palliative care

**DOI:** 10.1186/s12904-018-0272-x

**Published:** 2018-01-29

**Authors:** U. Olsson Möller, K. Stigmar, I. Beck, M. Malmström, B. H. Rasmussen

**Affiliations:** 1 Institute for Palliative Care, Lund University and Region Skåne, Lund, Sweden; 2 Faculty of Medicine, Department of Clinical Sciences Lund, Lund University, Lund, Sweden; 3 Faculty of Medicine, Department of Health Sciences, Lund University, Lund, Sweden; 4 Faculty of Health Science, The Research Platform for Collaboration for Health, Kristianstad University, Kristianstad, Sweden; 5 Department of Clinical Sciences Lund, Surgery, Lund University, Skåne University Hospital, Lund, Sweden

**Keywords:** Palliative care, Physiotherapy, Rehabilitation, Patient-centred care, Health care team, Qualitative research

## Abstract

**Background:**

A growing body of studies indicate benefits of physiotherapy for patients in palliative care, for symptom relief and wellbeing. Though physiotherapists are increasingly acknowledged as important members of palliative care teams, they are still an underutilized source and not fully recognized. The aim of this study was to explore the variety of activities described by physiotherapists in addressing the needs and problems of patients and their families in specialized palliative care settings.

**Methods:**

Using a free-listing approach, ten physiotherapists working in eight specialized palliative care settings in Sweden described as precisely and in as much detail as possible different activities in which patients and their families were included (directly or indirectly) during 10 days. The statements were entered into NVivo and analysed using qualitative content analysis. Statements containing more than one activity were categorized per activity.

**Results:**

In total, 264 statements, containing 504 varied activities, were coded into seven categories: Counteracting a declining physical function; Informing, guiding and educating; Observing, assessing and evaluating; Attending to signs and symptoms; Listening, talking with and understanding; Caring for basic needs; and Organizing, planning and coordinating. In practice, however, the activities were intrinsically interwoven. The activities showed how physiotherapists aimed, through care for the body, to address patients’ physical, psychological, social and existential needs, counteracting the decline in a patient’s physical function and wellbeing. The activities also revealed a great variation, in relation not only to what they did, but also to their holistic and inseparable nature with regard to why, how, when, where, with whom and for whom the activities were carried out, which points towards a well-adopted person-centred palliative care approach.

**Conclusions:**

The study provides hands-on descriptions of how person-centred palliative care is integrated in physiotherapists’ everyday activities. Physiotherapists in specialized palliative care help patients and families to bridge the gap between their real and ideal everyday life with the aim to maximize security, autonomy and wellbeing. The concrete examples included can be used in understanding the contribution of physiotherapists to the palliative care team and inform future research interventions and outcomes.

## Background

Patients in palliative care often experience high levels of physical and functional impairments related to the severity of the disease; in spite of this, physical therapy is often underutilized [1, 2]. A growing body of evidence shows that physiotherapy offers numerous benefits for patients with advanced life-threatening illnesses [2, 3]; however, poor access to physical therapy may jeopardize the potential benefit for patients and their families. The contribution of physiotherapy in palliative care may not yet be fully realized and a detailed description of the activities performed by physiotherapists (PTs) in relation to the patients’ and their families’ physical, psychological, social and existential needs may be useful.

Patients both with incurable cancer and with other, non-cancer diagnoses usually suffer from a high symptom burden such as fatigue, dyspnea, pain, lack of energy, weakness and appetite loss [4, 5]. With progressive disease, patients present with decreasing levels of physical function and mobility and diminished ability to perform activities of daily living [3, 6, 7], which can lead to distressing concerns about functional decline, uncontrolled symptoms and caregiver burden [8].

In palliative care, when attending to patients’ needs and concerns the main goal is to improve the quality of life for patients and their families.^1^ To assess and manage the characteristic multidimensional nature of suffering, defined as “total pain” [9], a multi-professional team, working in close collaboration, is needed. In several countries, Sweden included, self-referral to PTs is possible, which means that physiotherapy is accessible to all. However, in palliative care, self-referral is rare, and instead referral is often done by other palliative team members, usually registered nurses (RNs) [10]. Other health care staff (HCS) may be reluctant to refer patients with palliative care needs to physiotherapy because of the belief that PTs lack the necessary knowledge and skills required for palliative care, that they may foster false hope among patients and their families or because the HCS want to limit the number of professionals involved with the patient and their families [11–13]. This may hamper appropriate and equal access to physiotherapy, with poor identification of and response to the patients’ rehabilitative needs [3]. The close collaboration between the team members and the patients and their families therefore puts high demands on the transparency and understanding of the responsibilities, knowledge and skills of various professionals.

Physiotherapy in palliative care aims to maintain and/or improve physical function, minimize or eliminate complications and alleviate discomfort and pain [14–16]. It has been demonstrated that exercise in patients with palliative care needs is feasible [13, 17, 18] and a variety of appropriate physiotherapy techniques and treatments in palliative care have also been described for respiratory function, pain control and relaxation [15, 16, 19]. Physiotherapy interventions in patients with palliative care needs with various chronic conditions (cancer, chronic respiratory diseases, advanced heart failure, neurodegenerative disorders) have shown positive effects, with, for example, reduced pain [20–23], fatigue [23–27] and dyspnea [21, 23, 25, 26], and improved mobility and physical function [1, 22, 23, 25, 26, 28–30]. Physiotherapy interventions have also shown beneficial effects besides physical symptoms and function, with improvements in depression, anxiety and mood [23, 26] and improved wellbeing and quality of life [20, 22–24, 27].

From the patient’s perspective, physiotherapy has been reported in several studies to give a feeling of relief and wellbeing and hope of time-limited improvements in specific areas, which, as a result, may enable functional independence and the ability to continue being active [31, 32]. The patients in these studies have been reported to feel actively involved in both the planning and the performance of the physiotherapy, giving a sense of empowerment, control and improved quality of life [31, 32]. They recognized the importance of the PT’s role as tutor and motivator, giving the patient hope of improved physical function and quality of life [33].

The role of physiotherapy in palliative care has been described previously, on the one hand looking at traditional physiotherapy interventions and on the other, presenting the unique approach needed in the domains of end-of-life illness, dying and death [2, 3, 16, 34]. Physiotherapy in palliative care can have a preventive, educational and/or supportive function depending on the patient’s situation [35], and depending on factors such as a patient’s sudden deterioration, priority given to other therapies, and conflicting interests [6]. The diverse and complex interventions required by patients and their families with palliative care needs from the point of view of PTs daily work have, to our knowledge, not previously been explored extensively. A detailed description of the variety of common physiotherapy interventions can enlighten the complexity of the actions taken to minimize the negative effects of the disease and/or its treatments and enhance the awareness of PTs’ contribution within the palliative care team. The description may contribute to the knowledge on how to address the patients’ and their families’ physical, psychological, social and existential needs.

## Methods

### Aim

To explore the variety of activities described by physiotherapists in addressing the needs and problems of patients and their families in specialized palliative care settings.

### Setting

The study was performed in eight specialized palliative care (SPC) settings in two county councils in the south of Sweden, with 0.4–2.5 physiotherapy posts per setting. Five SPC settings had a palliative care ward with eight to 29 beds and one unit had five beds in a medical ward. All units had advanced home care services with the capacity to manage 20–50 patients. The palliative care teams consisted of PTs, occupational therapists (OTs), physicians, RNs, nurse assistants, social workers, nutritionists and deacons or priests (all herein defined as “health care staff [HCS]”). In 2015, approximately 75% of the patients had a cancer diagnosis and the remaining patients suffered from neurological, respiratory or cardiovascular diseases [36]. The mean length of stay in the palliative care wards in the region in 2016 was 14.8 days and in the palliative home care settings 57.5 days [37] . The majority of patients’ in the palliative care wards were in the dying phase.

### Participants

All twelve PTs working in the participating settings were asked to participate in the study and ten agreed. Of these PTs, one worked full-time in a palliative care ward, four worked exclusively in the advanced home care service, and the others combined working at the ward with working in the home care service. Nine worked full-time and one worked 25% in SPC. Their work experience as PTs ranged from 3 to 41 (median 18) years and they had worked in SPC from 6 months to 15 years (median 2.5 years). All had had supplementary education and training in, for example, palliative care, acupuncture, complex decongestive therapy (CDT), transcutaneous electrical nerve stimulation (TENS), respiratory treatments or tutoring.

### Data generation

The study utilized an anthropological free-listing technique for data collection previously used to describe non-pharmacological caregiving activities (NPCAs) in SPC settings [38]. A pilot study to test the feasibility of the data collection procedure was conducted, in which the ten PTs were requested to write down interventions and activities performed during one day, in which patients and their families were included (directly or indirectly). They were asked to describe these as precisely and detailed as possible using everyday language. The PTs then met with a researcher (U.O.M.) to discuss the written notes and reflect upon practical or methodological difficulties in clarifying and improving data collection, for example by giving more detailed descriptions. Thereafter, the PTs continued to write down statements during 10 self-selected days over a 3-month period (December 2015 to February 2016). To understand variation rather than frequency of occurrence, the PTs consecutively documented new but not repeating reoccurring activities. Data from the pilot study were included in the final analysis.

### Data analysis

**Fig. 1:**
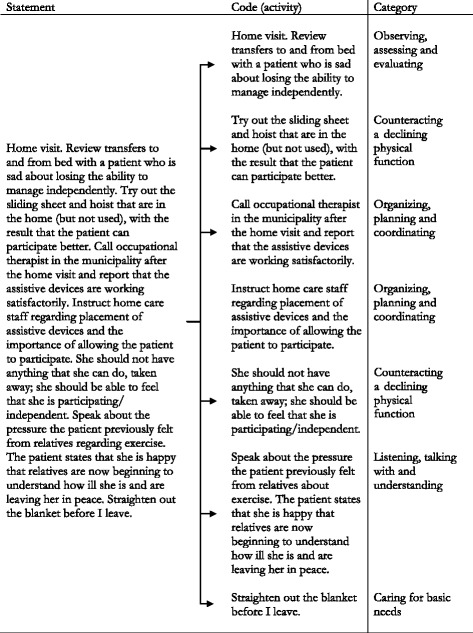
The process of dividing composite statements into separate activities, and further sorting them into separate categories

All unedited free-listing statements were entered into NVivo version 10, 2012 (NVivo qualitative data analysis software, QSR International Pty Ltd., Doncaster, MD, USA). Statements were analysed using qualitative content analysis [39] and employing an inductive approach, i.e. coding and category development were driven by the content of the written activities. The vast majority of the statements were composites, consisting of more than one activity. All authors discussed the statements, to identify, define and distinguish between the activities in each statement. The composite statements were coded for each activity, and have therefore generated multiple codes (see Fig. 1). The codes were sorted into content groups (categories), which were labelled. Where applicable, an activity was attributed to more than one category. All authors met twice to in depth discuss the content, sorting and labelling of the categories. To increase the validity, member-check [40] of the content and labelling of the categories was performed by three of the participating PTs. The labelling of the categories and the development of a matrix was inspired by Lindqvist et al. (2012) [38], who report on the complex composite nature of the recorded statements. One coding dimension in the matrix consists of the categories describing physiotherapy activities. The second coding dimension describes who was involved in the activities, that is, the patients, family or HCS (see Table 1).

**Table 1:** Matrix overview of relative frequencies of codes in each category, by type of activity and recipient of the activity

	Patients (70%*)	Families (11%*)	HCS (19%*)
Counteracting a declining physical function	20%	2%	1%
Informing, guiding and educating	18%	31%	17%
Observing, assessing and evaluating	18%	1%	3%
Attending to signs and symptoms	17%	1%	0%
Listening, talking with and understanding	11%	45%	2%
Caring for basic needs	11%	7%	0%
Organizing, planning and coordinating	5%	13%	77%
	100%	100%	100%

*of analysed statements (*N* = 504). HCS = health care staff

## Results

In all, 264 statements were recorded and several of them were in the form of a composite statement containing 506 varied activities in total. Two activities were verbatim repetitions and were excluded from the analysis. From the 504 activities, we created seven categories (see Table 1).

Although the categories of activities are not mutually exclusive, in the presentation below we describe them separately for the sake of clarity. The categories are ordered by number of activities that involved patients (see Table 1). Table 2 presents the coding matrix, with illustrative examples.

**Table 2:** Matrix of categories, with examples of coded statements

	Patients	Families	HCS
Counteracting a declining physical function	Exercise with patient for retained walking ability and ability to get up.	Conversation with patient and family about what daily life is like and what could make things easier for them.	Educate HCS regarding placement of assistive devices and the importance of allowing the patient to participate.
Informing, guiding and educating	Give tips about posture and resting positions when sitting/lying.	The patient’s family members also participate and discuss safety/security during mobilization.	Educate staff at the residence about ALS, along with nurse and dietitian, followed by a question and answer session regarding the patient’s symptoms.
Observing, assessing and evaluating	Observe how the patient manages transfers, patterns of movement and balance when she comes into the room where I am waiting.	Family members visit and we discuss how things are going in the home.	Together with ward staff, see how things are going with transfers for newly admitted patient.
Attending to signs and symptoms	Introduce breathing exercises – using the PEP valve for more efficient breathing when the patient uses shallow and high costal breathing.	Show daughter lymphatic drainage to relieve pain.	–
Listening, talking with and understanding	Speak about the pressure the patient previously felt from relatives about exercise.	Meet a family member of a patient who is sad. We hug and she says that her mother is dead. Provide comfort and support.	One of our patients has died and we talk about this.
Caring for basic needs	The patient falls asleep during treatment and wants to remain in bed afterwards, I help her get comfortably straightened out and leave the window slightly open before I go.	Help relative with kitchen chores because he is having difficulty with transfers.	–
Organizing, planning and coordinating	Home visit together with patient for the purpose of increasing quality of life and preparing for returning home and discharge to ASIH.	Phone call with family makes me schedule a home visit together with the assistive technology consultant.	Contact nurse regarding family’s questions about samples and blood transfusion.

*ALS* amyotrophic lateral sclerosis, *ASIH* Avancerad sjukvård i hemmet (Advanced health care at home), *HCS* health care staff, *PEP* positive expiratory pressure

### Counteracting a declining physical function

In this category, the activities directed to the patient had a focus on facilitating good conditions for human movement. The patients were given practical tools to be able to regain and/or maintain physical function and desired functional independence including in the dying phase. Activities to counteract a declining physical function included using sliding sheets to manage turning around in bed, using stocking aids in order to avoid having to rely on, the not wish for, home care services, or using a wheelchair or walker to be able to move around.

The physiotherapy activities could include a mix of physical activity and exercise aiming to accommodate various needs (physical, psychological, social or existential) and could, for example, combine a walk with a wheelchair and walking exercise when going with the patient to a workplace visit (see Fig. 1 for the interwoven nature of some activities). Other activities described included traditional physiotherapy in palliative care, such as strength, flexibility, balance, and functional exercises, some of them aimed at safety aspects such as safe transfers to reduce falls and fear of falling, for example by introducing a rolling walker.

### Informing, guiding and educating

Words such as “informing”, “instructing”, “guiding”, “advising” and “educating” predominated in the descriptions of activities in this category, which related to patients and their families as well as the HCS and mainly aimed at self-management. For patients and families, the activities included information about the performance and safety aspects of transferring or technical instructions in how to apply and use aids such as TENS, compression stockings, orthoses, and medical devices. Information could also aim to prevent symptoms, for example a patient might be informed about an elevated leg position and active feet and leg exercises to prevent oedema while another patient might be instructed in how to adequately move her arm to prevent pain. Families were guided in how to alleviate the patient’s symptoms by optimizing transfer techniques when supporting the patient in performing exercise or relaxation, or by giving massage.

The activities included guiding the patient in how to balance activity and energy expenditure, on the one hand, and fatigue, on the other, taking their wishes and priorities as point of departure. The patients sometimes also turned to the PTs for advice. For example, a patient might consult a physiotherapist for support when in disagreement with her family about her preferred activity level. Guiding the patient could also include encouraging them to continue to be active or demonstrating a physical improvement.

Activities in this category also included educating SPC team members or HCS in other health care organizations (for example municipal elderly care). Some activities were generic in nature, such as instructing the patient on the use of a new aid, but most aimed to provide specific information and instructions in relation to a certain patient: for example, to maintain the patient’s functional independence by placing aids at the right place or enabling pain relief by instructing adequate resting positions.

### Observing, assessing and evaluating

What PTs observed, assessed and evaluated most often included physical function and symptoms such as pain, oedema, and breathlessness. The assessment at home was described as a “scanning procedure”, an observation of the patient’s functional status and the patient’s surroundings to identify situations where the patient was in need of support. Both the patient and their family could be asked to describe to the PTs how the patient was able to move around at home or in the near environment, giving a picture of the difficulties and possibilities. The assessments to identify the patient’s problems and needs could be verbal (for example, taking the patient’s medical history and establishing their current activity level), visual (assessing the patient’s movements, and their environment) or manual (palpation). Sometimes the PTs described abstaining from making an assessment if causing pain or if the patient was too tired. Requests for assessment came from the other team members as well as from the patients or their families. Assessments were sometimes performed together with other team members.

Evaluations of various activities, done by phone or during home visits, included outcomes such as pain relief, reduced limb circumference (oedema) or follow-up of an exercise programme, but mainly focused on how the patient managed everyday life activities. Sometimes the PTs just stayed in contact to check if everything was alright. Activities related to goal setting, other than symptom relief, were performed to try to meet patients’ wishes for increased independence or a general sense of wellbeing, for example by taking a walk outside.

### Attending to signs and symptoms

The activities in this category were directed solely to patients, aiming to treat and relieve a variety of symptoms such as pain, dyspnea, oedema, fatigue and anxiety. The PTs described many aids and techniques such as breathing techniques, TENS, acupuncture, compression stockings, passive and active movement and CDT. These treatments were mainly given to treat one symptom. A home visit could, however, include various treatments targeting different symptoms, for example TENS for pain relief, positive expiratory pressure (PEP) for ventilation, and leg exercises for increased flexibility. The majority of the activities described hands-on treatments; others referred to the introduction of various aids such as neck collars or therapeutic beds.

### Listening, talking with and understanding

The activities in this category related to communication, mainly between patients and PTs, but also between the patients’ families and PTs, with focus on “being there”, listening to, talking with and trying to understand the patients’ and families’ situation. PTs described listening to the patients’ fears, worries and concerns such as fear of loss of function or independence, or their expressions of death wishes, but also feelings of gratitude and expressions of positive expectations and wishes for the future. The conversations sometimes replaced a planned activity which was then postponed because the patient had a greater need to talk. Other activities relating to communication, often described as “small talk”, were also described, such as when engaging in joyful conversation about life events, vacation trips, gardening, food, the weather or favourite interests; sometimes these took place over a cup of coffee.

Several of the described activities included listening and talking to families without the patient being present, such as giving comfort and support to worried and sad families during and after the episode of care. In some cases, the activities were also described as bereavement support as a planned activity or as team conversations to review the care of the patients who had recently died.

### Caring for basic needs

The majority of the activities in this category related to patients in the dying phase. Caring for basic needs included providing functional support when the patient went to the bathroom or giving help in dressing, or activities that aimed at providing general relief and a sense of wellbeing, for example therapeutic massage, helping the patient to find a comfortable resting position or sit on the bedside.

Another perspective of basic caring was described in activities such as putting on the patient’s favourite music, solving a crossword puzzle together, opening a window or adjusting a blanket and making sure the room looked nice. One of the participants described transferring a patient to a wheelchair and offering him his favourite ice cream in the unit dining hall and sharing some of it with him. The activities also included activities directly targeting families, for example minor repair of aids, helping out with kitchen chores or feeding the dog – in other words, doing a “little extra” for the patients and their families.

### Organizing, planning and coordinating

In this category, the PTs described organizing, planning and coordinating activities within the SPC team, between the SPC team and the patients and their families, or with HCS in various health care organizations. The patients’ often rapidly changing condition characterized this category. Basically, activities with patients were planned a few days ahead, including those that were related to a patient’s discharge from the hospital, planned team home visits, or follow-up telephone calls. The planned activities also included medical, ethical or social team meetings. The flexible way of working and the close collaboration between the SPC team members was made evident in the everyday planning of the PT’s work, for instance when a planned home visit by the PTs was reorganized to a team visit (with the PTs, RNs, OTs and/or physicians) in response to a patient’s rapidly increasing sense of pain. On another occasion, planned activities were rescheduled because of an acute visit to a patient after a fall.

Even transdisciplinary activities were described, often done as a consequence of changes to planned activities. PTs might perform, as a part of a planned visit, minor wound care or might remove expired medications to cover for a temporary shortage of RNs, or deliver and adjust an aid, an activity usually performed by OTs. The opposite situation was also described by the PTs, when RNs delivered an extra pair of compression stockings or a PEP device.

The activities described in this category also related to coordinating care with HCS in other health care organizations, such as making telephone calls or holding meetings to report the status of a patient, or for general or specific learning opportunities. The PT’s coordinating role was also described as providing a link between the patient, their family, and other HCS, aiming to solve the patients’ problems by explaining something to them from the PT’s professional point of view or by actively trying to make things happen.

## Discussion

The described activities revealed a great amount of variation in the work of PTs in SPC, in relation not only to *what* they do, but also to *why, how, when, where*, *with whom* and *for whom* the activities are carried out. Holistically, the what and the why, how, when, etc. are inseparable. In this paper, in an attempt to explore and clarify physiotherapy in SPC, we have chosen to categorize the activities; however, in practice the activities are intrinsically interwoven to address the patient’s and their family’s physical, psychological, social and existential needs. The activities described above aim to improve and/or maintain patients’ and their families’ independence and wellbeing, and an underlying intention with many of the activities may be seen as helping and supporting patients and their families to bridge the gap between the real and the ideal everyday life. Several studies have identified that unmet needs and loss of ability to manage daily living causes much distress to severely ill patients [41, 42]. By counteracting the physical decline and diminishing the gap between what the patient wants to and can do, the activities appear to give patients opportunities – some of the once taken-for-granted freedom – to decide when, where and how to perform everyday activities and thus regain a sense of dignity, autonomy and control.

The described activities are examples of how PTs, through care of the body, aim to meet patients’ physical, psychological, social and existential needs. Physical activity and exercise may target physical achievements so as to maximize independence, but they may also provide momentary wellbeing, for example by affecting the mind through bodily movement. For instance, linking a walking exercise to a visit to the workplace or to the mailbox can become a meaningful activity for the patient as it may relate to the previous self and everyday life. Thus, many of the described activities appear to be person-centred and holistic [38, 43] by integrating the means to an end with the end in itself. Describing the intention behind the activity is important in clarifying the PT’s role in SPC.

When independence and self-management are no longer attainable (as for patients in the dying phase), the activities seem to be geared towards wellbeing and comfort rather than rehabilitation. In other words, there is a change from a rehabilitative to a supportive approach. These findings endorse the models for physiotherapy practice for patients with palliative care needs, described by Briggs (2000) [34]. Briggs describes six models, Traditional rehabilitation; Rehab light; Rehabilitation in reverse; Case management; Skilled maintenance; and Supportive care, the first two of which mainly focus the early palliative phase*.* He argues that in practice, there may well be a shift from one model to another, with several models sometimes used concurrently [34]. Our study underpins and clarifies the complex integration of activities and the changing approach needed to address patients’ and families’ needs.

One of the prerequisites for PTs in SPC to be able to provide care to patients with palliative care needs was to engage in close and flexible collaboration with team members, other HCSs, non-professional caregivers and health care organizations. In these data, the PTs had an active coordinating role in the team, which became evident when PTs responded immediately to patients’ rapidly changing situation, by reorganizing planned team activities. Organizational and practical requirements also included transdisciplinary activities and this way of working challenges the boundaries between different professions, that is, the interaction between the “specific” and the “general”. Some “general” activities were described, such as combing a patient’s hair or giving the patient a glass of water, putting on a patient’s favourite music, adjusting a blanket or opening a window. Such activities are an important part of palliative care [38] and are in line with the description of the role of PTs in SPC as a “specialist generalist”, i.e. “going beyond the symptom to explore the wider concept of what it means to each patient” [44] and supporting the holistic, often complex needs of patients in palliative care. Utilizing a holistic approach to care, PTs also engaged in patients’ and families’ everyday life, for example by engaging in small talk or having a cup of coffee, and gave emotional support to the family to support them in dealing with their own emotions. However, these activities are presumably performed by all HCS and non-professional caregivers, and are a requirement of person-centred palliative care [38]. To clarify daily practice, future studies should explore the general and specific activities performed by various HCS and non-professionals caregivers in SPC to better understand the optimal allocation of HCS resources.

Participating PTs mainly worked in the specialized palliative home care service and therefore most activities in this study were performed and described in this setting. Care at home opens up for a somewhat different approach when observing, assessing and treating the patient’s problems. Instead of assessing and treating a specific symptom or disability, there is a so-called “scanning procedure” at home that enables a comprehensive intervention in the patient’s and their family’s everyday context. This sometimes preventive and often practical approach enables instant problem solving in everyday life; also, it allows the patient to remain as safely active as possible, again aiming to support the patient and families to continue to live their lives with some semblance of normality (from their point of view). Our results support the findings of Klarare et al.’s (2017) [45] study on patients’ and families’ narratives about SPC home teams, where PTs’ activities (related to practical aspects in the home environment) fostered a feeling of security. Security at home, achieved by the SPC home team as being present and competent, enables patients and families to continue living everyday life and at the same time prepare for death [46]. However, the available evidence regarding home-based SPC is still nascent and more studies in the area are needed [47].

In patients with serious illness, various forms of physiotherapeutic interventions are needed to motivate and engage patients [48]. Our study reinforces complexity and interwoven nature of physiotherapy in palliative care and the challenges of shaping and directing experimental research. It may be feasible to create an experimental study, for example, about the effect on and experiences of independence when using a walker or pain relief after TENS. What our findings highlight is the necessity of including not only physical target outcomes (frequency, intensity and duration) but also psychological, social and existential outcomes such as dignity, hope and meaning.

This study supports the findings of previous research on the role of physiotherapy in palliative care, but it also adds to the knowledge by enlightening the variation, breadth and complexity of physiotherapy in daily practice. The described activities being performed by PTs in SPC point towards a well-adopted person-centred palliative care approach [38, 43] and show that physiotherapy is an essential piece in the puzzle of addressing patients with palliative care needs, and their families. Further studies to explore activities performed by PTs in various health care contexts and in relation to other HCS and non-professional caregivers are warranted to further explore, compare and describe physiotherapy in SPC.

### Methodological considerations

This study does not claim to provide a comprehensive description of physiotherapy in SPC, but should be seen as a contribution to the clarification of PTs’ activities in SPC. Obviously, the descriptions are context-specific and consequently have some limitations. The study was performed in a part of Sweden where specialized palliative home care is fully developed [49, 50] and where the physiotherapy profession is quite well established within the SPC teams. But, for example, group exercise classes, sometimes used as a treatment option, were not performed in the participating settings, and this is one of several factors affecting the content of the descriptions. The small number of PTs included may be considered as a limitation; however, they represent eight out of nine palliative care settings in two county councils and a pilot study was performed to test and clarify the data generation process. The free-listing approach also made it possible to explore physiotherapy in detail from a daily practice perspective, uncoloured by researchers’ assumptions. Another concern may be that the study did not specify activities related to the patients’ diagnoses, and in the participating units approximately 75% of the patients had a cancer diagnosis. However, as worldwide mainly patients with a cancer diagnosis are referred to SPC, this factor may not limit transferability.

## Conclusions

This study contributes to the knowledge of physiotherapy in SPC and of how person-centred palliative care is integrated in everyday PTs’ activities. Data show that various physiotherapy activities are complex and inextricably linked to each other, resulting in physiotherapy treatments that are meaningful to patients, thus aiming to bridge the gap between what the patients wants to do and what they can do. The concrete examples in this paper can be used in understanding the contribution of physiotherapy to the palliative care team in the education of PTs and other health care staff. The detailed description can also inform future research interventions and outcomes. Further studies in the daily practice of palliative care are warranted.

## Abbreviations

CDT: Complex decongestive therapy
HCS: Health care staff
NPCA: Non-pharmacological caregiving activity
OTs: Occupational therapists
PEP: Positive expiratory pressure
PTs: Physiotherapists
RNs: Registered nurses
SPC: Specialized palliative care
TENS: Transcutaneous electrical nerve stimulation

## Footnotes

1 We use the term “families” in its broadest sense, to mean all significant others.
